# Modulation of the Oxidative Stress and Lipid Peroxidation by Endocannabinoids and Their Lipid Analogues

**DOI:** 10.3390/antiox7070093

**Published:** 2018-07-18

**Authors:** Cristina Anna Gallelli, Silvio Calcagnini, Adele Romano, Justyna Barbara Koczwara, Marialuisa de Ceglia, Donatella Dante, Rosanna Villani, Anna Maria Giudetti, Tommaso Cassano, Silvana Gaetani

**Affiliations:** 1 Department of Physiology and Pharmacology “V. Erspamer”, Sapienza University of Rome, Piazzale Aldo Moro 5, 00185 Rome, Italy; cristinaanna.gallelli@uniroma1.it (C.A.G); silvio.calcagnini@uniroma1.it (S.C.); adele.romano@uniroma1.it (A.R.); justynabarbara.koczwara@uniroma1.it (J.B.K); marialuisa.deceglia@uniroma1.it (M.d.C.); donatella.dante@uniroma1.it (D.D.); silvana.gaetani@uniroma1.it (S.G.); 2 C.U.R.E. University Centre for Liver Disease Research and Treatment, Department of Medical and Surgical Sciences, Institute of Internal Medicine, University of Foggia, 71122 Foggia, Italy; rosanna.villani@unifg.it; 3 Department of Biological and Environmental Sciences and Technologies, University of Salento, Via Monteroni, 73100 Lecce, Italy; anna.giudetti@unisalento.it; 4 Department of Clinical and Experimental Medicine, University of Foggia, Via Luigi Pinto, c/o Ospedali Riuniti, 71122 Foggia, Italy

**Keywords:** oxidative stress, lipid peroxidation, reactive aldehydes, reactive oxygen and nitrogen species, free radicals, endocannabinoids, cannabinoid receptors, peroxisome proliferator-activated receptors, transient receptor potential vanilloid, G protein-coupled receptors

## Abstract

Growing evidence supports the pivotal role played by oxidative stress in tissue injury development, thus resulting in several pathologies including cardiovascular, renal, neuropsychiatric, and neurodegenerative disorders, all characterized by an altered oxidative status. Reactive oxygen and nitrogen species and lipid peroxidation-derived reactive aldehydes including acrolein, malondialdehyde, and 4-hydroxy-2-nonenal, among others, are the main responsible for cellular and tissue damages occurring in redox-dependent processes. In this scenario, a link between the endocannabinoid system (ECS) and redox homeostasis impairment appears to be crucial. Anandamide and 2-arachidonoylglycerol, the best characterized endocannabinoids, are able to modulate the activity of several antioxidant enzymes through targeting the cannabinoid receptors type 1 and 2 as well as additional receptors such as the transient receptor potential vanilloid 1, the peroxisome proliferator-activated receptor alpha, and the orphan G protein-coupled receptors 18 and 55. Moreover, the endocannabinoids lipid analogues *N*-acylethanolamines showed to protect cell damage and death from reactive aldehydes-induced oxidative stress by restoring the intracellular oxidants-antioxidants balance. In this review, we will provide a better understanding of the main mechanisms triggered by the cross-talk between the oxidative stress and the ECS, focusing also on the enzymatic and non-enzymatic antioxidants as scavengers of reactive aldehydes and their toxic bioactive adducts.

## 1. Introduction

Oxidative stress and lipid peroxidation are the consequences of a deregulated redox homeostasis that results in the accumulation of highly reactive molecules and cellular injury, especially in those tissues with a high oxygen consumption, such as heart, kidney, and brain, thus leading to cardiovascular [1,2], renal [3], and neurodegenerative diseases [4,5,6], just to mention a few. Examples of the possible repercussions of free radical damage are provided in this review with special emphasis on lipid peroxidation-derived reactive aldehydes including acrolein (ACR), malondialdehyde (MDA), and 4-hydroxy-2-nonenal (4-HNE), among others [7].

To get a deeper insight into the cellular pathways that regulate reactive oxygen and nitrogen species (ROS/RNS) as well as reactive aldehydes formation, there is a growing interest in identifying free radical scavenging molecules that can prevent cell death following oxidative stress-induced damage of cellular membranes. In this perspective, over the last few years, the endocannabinoid system (ECS) has attracted significant attention because of the existing cross-talk between endocannabinoids (ECs) as well as their lipid analogues and various redox-dependent processes. Therefore, the pathways by which the ECs and their lipid-related mediators contribute to the modulation of oxidative stress and lipid peroxidation represent a significant research area that will yield novel pharmaceutical strategies for the treatment of diseases characterized by a redox imbalance.

The cannabinoid receptors type 1 (CB1) and 2 (CB2), together with additional ECs receptor targets, take part in the complex ECS and, because of their wide distribution, they may play a role in mediating the antioxidant properties of ECs [8,9,10]. However, the great diversity of results in this field discloses the requirement of a better understanding on the pathways by which these receptors are involved in regulating oxidative stress and lipid peroxidation processes.

In this review, we will provide an overview of the role of the ECS in pathological conditions related to a redox status imbalance, leading to a better comprehension of the intricate routes that are associated to the antioxidant properties exerted by the ECs, thus enhancing the research in finding a therapeutic benefit for cannabinoid-based drugs in various redox-dependent disorders.

## 2. Oxidative Stress and Lipid Peroxidation

Oxidative stress can be described as an imbalance between the production of oxidant species and the antioxidant defenses, which may affect cellular redox homeostasis leading to molecular alterations and thus resulting in cell and tissue damage [11]. The term “oxidants” is a general term used to identify several groups of reactive molecules among which ROS and RNS are considered the most interesting from a biological point of view. ROS/RNS are natural byproducts of aerobic metabolism and are produced by all living multicellular organisms. ROS include free oxygen radicals and non-radical molecules, such as superoxide anion (O_2_•^−^), hydroxyl (•OH), peroxyl, alkyl, and alkoxyl radicals, as well as singlet oxygen (^1^O_2_), hydrogen peroxide (H_2_O_2_), ozone (O_3_), and hypochlorous acid (HClO), while RNS include nitrogen compounds such as nitric oxide (•NO), nitrogen dioxide (NO_2_•), nitrate (NO_3_^−^), nitrite (NO_2_^−^), and peroxynitrite (ONOO^−^) [12,13].

In mammals, the main cellular sources of ROS/RNS are the mitochondrial and microsomal electron transport chains [14], the NADPH oxidase enzymes (NOXs), which consist of seven isoforms with various tissue distributions and mechanisms of activation [15,16], the flavoenzyme endoplasmic reticulum oxireductin 1 [17], nitric oxide synthase (NOS) [18], cytochrome P450 enzymes [19], cyclooxygenases (COXs), lipoxygenases (LOXs) [20], xanthine oxidase [21], diamine oxidase [22], and prostaglandin synthase [23]. In addition to these endogenous sources, the ionizing radiation, ultraviolet rays, pathogens, xenobiotics (e.g., drugs, herbicides, fungicides, trace metals, etc.), and environmental pollutants (e.g., smog, cigarette smoke, smoke from wood combustion, etc.) are identified as exogenous sources of ROS/RNS [24], which may seriously alter the fundamental oxidants-antioxidants balance.

To date, growing evidence confirms that ROS/RNS are produced by healthy cells in a highly regulated fashion in order to maintain the intracellular redox homeostasis. Moreover, ROS/RNS regulate several cellular functions ranging from immune defense to gene expression regulation, thus acting as reactive molecules secreted against circulating pathogens [25] or as second messengers of specific signaling pathways [26]. The crucial role played by ROS/RNS in immune defense was demonstrated by the discovery of the chronic granulomatous disorder (CGD), a hereditary disease characterized by NOX type 2 (NOX2)-defective phagocytes [27] which are unable to produce ROS/RNS. This genetic defect leads CGD patients in developing a primary immunodeficiency due to the inability of host innate defense to kill and digest ingested pathogens such as bacterial and fungal cells [28,29,30,31]. Moreover, ROS/RNS play also an important role in the cardiovascular system because of their ability to regulate blood pressure. In particular, the endothelial NOX2 isoform regulates the release of •NO, the endothelium-derived relaxing factor, which modulates the caliber of blood vessels, through the production of O_2_•^−^. In hypertension and other vascular pathologies, NOX2 seems to be up-regulated leading to a reduced •NO bioavailability and to the consequent oxidants-antioxidants imbalance in the endothelium, further worsening the oxidative state [32,33,34]. Moreover, in vivo studies of single nephron function and in vitro studies performed on perfused juxtaglomerular apparatus preparation demonstrated that also the normal renal functions are modulated by ROS/RNS. In particular, O_2_•^−^ and •NO, which are generated by NOX type 3 (NOX3) and NOS type 1 (NOS1) enzymes, respectively, modulate afferent arteriolar tone and control Na^+^ reabsorption and renal oxygenation by regulating the tubuloglomerular feedback response [35,36,37]. Furthermore, in the loop of Henle, ROS/RNS increase the absorption of NaCl by modulating the activity of the Na^+^/H^+^ exchanger [38,39]. In airway and pulmonary artery smooth muscle cells of the lung, NOX2-generated ROS/RNS act as signaling intermediates, which regulate the proliferation and differentiation by the activation of the nuclear factor-κB (NF-κB) and NOS2, and they further show an important role in O_2_ sensing [39,40,41].

Moreover, ROS/RNS formation by mucosal cells of the colon seems to modulate the serotonin production by enterochromaffin cells through a NOXs-dependent system, thus contributing to the regulation of serotonin secretion as well as intestinal motility [42]. ROS/RNS have also a fundamental role in the central nervous system (CNS), in particular in central autonomic neurons. To this regard, ROS/RNS produced by NOX2 in the nucleus of the solitary tract, in the hypothalamic paraventricular nucleus, and in the subfornical organ modulate angiotensin II signaling, thus contributing to the regulation of cardiovascular homeostasis [43,44]. Moreover, in microglia but not in astrocytes, H_2_O_2_ formation by NOX2 enzyme is involved in the regulation of cell proliferation [45].

Beyond the role as signaling molecules, it has been shown that the aberrant ROS/RNS formation is the leading cause of cell and tissue oxidative stress-induced damage. Indeed, it is well known that excessive levels of ROS/RNS may directly damage lipids containing carbon-carbon double bounds such as cholesterol, glycolipids, phospholipids, and polyunsatured fatty acids (PUFAs), which are abundant within cellular membranes. To this regard, free radical–mediated lipid peroxidation of PUFAs is one of the main mechanisms by which ROS/RNS induce the generation of reactive aldehydes [46]. Due to their abundance of reactive hydrogens, PUFAs are more oxidation-prone lipids compared to monounsatured fatty acids. PUFAs include the ω-3 (e.g., linolenic acid, eicosapentaenoic acid and docosahexaenoic acid) and ω-6 (e.g., linoleic acid and arachidonic acids) fatty acids.

**Figure 1:**
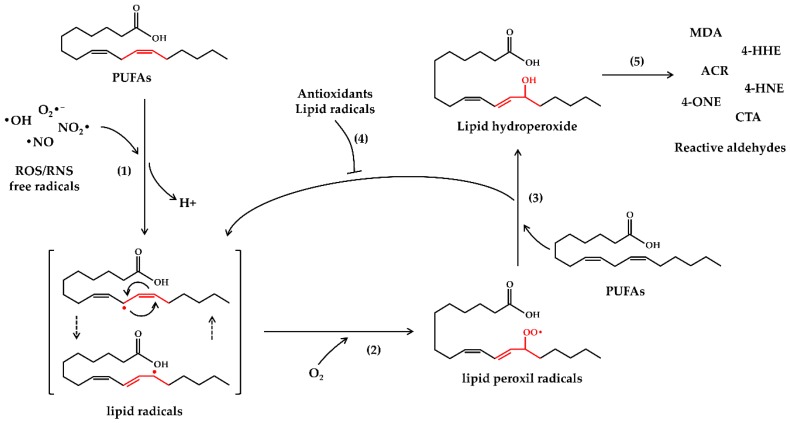
Schematic diagram of the free radicals-mediated peroxidation of polyunsatured fatty acids (PUFAs). ROS/RNS: reactive oxygen and nitrogen species; ACR: acrolein; MDA: malondialdehyde; CTA: crotonaldehyde; 4-HNE: 4-hydroxy-2-nonenal; 4-HHE: 4-hydroxy-hexanal; 4-ONE: 4-oxo-nonenal. During the initiation phase (1), ROS/RNS free radicals react with PUFAs and rip off an allylic hydrogen thus forming lipid radicals. Generally, lipid radicals tend to be stabilized by a molecular rearrangement. (2) In the propagation phase, lipid radicals react with oxygen to form lipid peroxyl radicals, which in turn react with PUFAs or other nearby lipids resulting in the formation of new lipid radicals and lipid hydroperoxides (3). During the termination phase (4), antioxidants or lipid radicals block the propagation phase by donating a hydrogen atom to lipid peroxyl radicals resulting in the formation of non-radical products. Nevertheless, lipid hydroperoxides are highly unstable therefore they are further degraded into reactive secondary products such as ACR, MDA, 4-HNE, and other reactive aldehydes (5).

Lipid peroxidation is a chain reaction, which, once started, proceeds through three main steps referred to initiation, propagation and termination [47]. Moreover, lipid peroxidation may occur by several mechanisms: (1) free radical-mediated oxidation [47], (2) enzymatic oxidation, and (3) spontaneous oxidation [48]. In this review, we will focus mainly on the free radical–mediated mechanisms that lead to the formation of reactive aldehydes from PUFAs. In particular, the free radical-mediated oxidation of PUFAs occurs through the following reactions: (1) During the initiation phase, ROS/RNS free radicals attack PUFAs ripping off one hydrogen atom, leading to lipid radicals formation. (2) During the propagation phase, lipid radicals react with oxygen molecules, thus producing peroxyl radicals, which, in turn, react with nearby lipids resulting in the formation of new lipid radicals and lipid hydroperoxides. Due to their high instability, lipid hydroperoxides are further degraded into reactive secondary products, such as ACR, MDA, 4-HNE and other reactive aldehydes [7]. (3) During the termination phase, peroxyl radicals may react with other radicals thus generating less reactive compounds, which block the propagation phase (Figure 1) [49].

Today, it is well accepted that oxidative stress and lipid peroxidation are key features in the pathogenesis of several disorders. Indeed, it has been reported that lipid peroxidation products may interfere in vivo with several biological processes, such as substrate-receptor interaction, signal transduction, gene expression, and homeostatic responses to intracellular and environmental stimuli [50,51,52,53]. Currently, the main objective of research focused on oxidative stress, lipid peroxidation, and reactive aldehydes is the characterization of the pathogenic mechanisms in several disorders as well as the identification of specific biomarkers for diseases.

**Figure 2:**
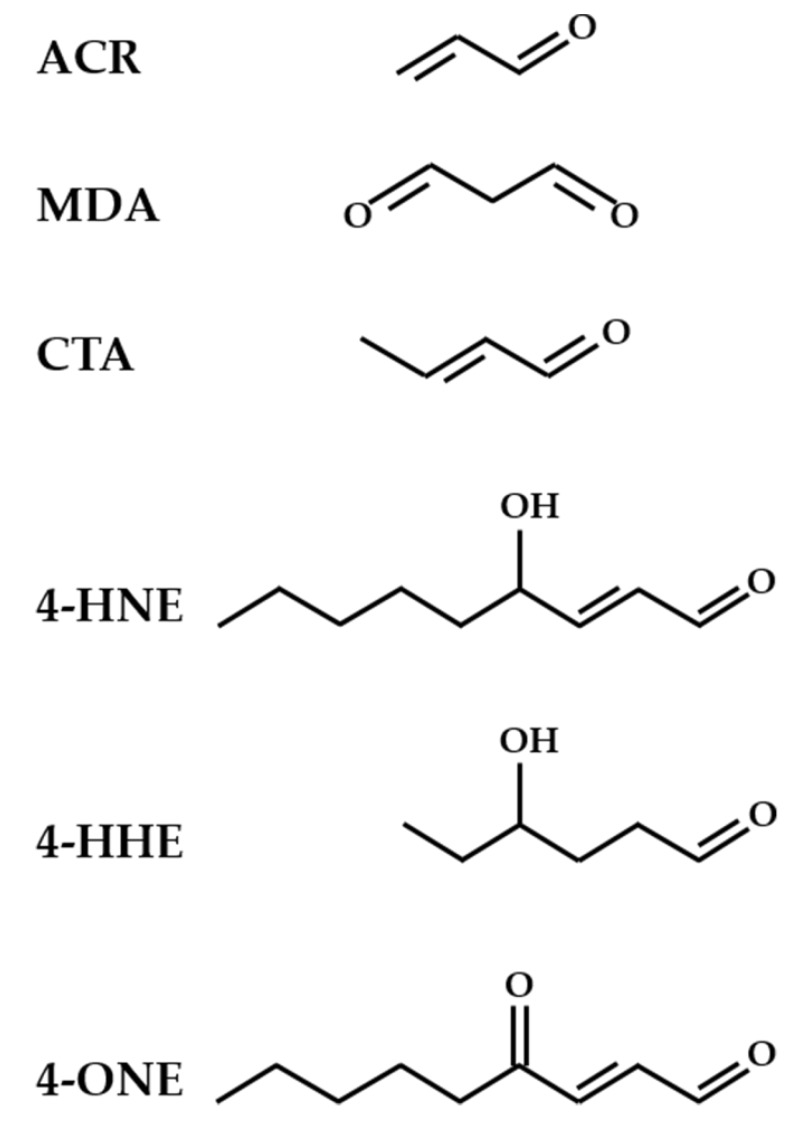
Chemical structures of the main reactive aldehydes produced by lipid peroxidation. ACR: acrolein; MDA: malondialdehyde; CTA: crotonaldehyde; 4-HNE: 4-hydroxy-2-nonenal; 4-HHE: 4-hydroxy-hexanal; 4-ONE: 4-oxo-nonenal.

Among the reactive aldehydes, the most frequently studied are ACR, MDA, 4-HNE, 4-hydroxy-hexanal (4-HHE), 4-oxo-nonenal (4-ONE), and crotonaldehyde (CTA) (Figure 2).

**Figure 3:**
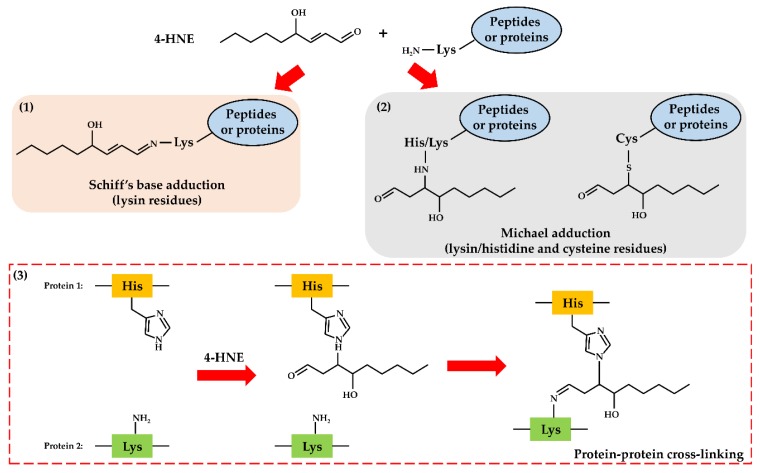
Schematic representation of protein adducts formation and protein-protein cross-linking by 4-HNE. Reactive aldehydes are able to modify peptides/proteins by the formation of toxic adducts which may alter the structure and/or the function of targeted peptides/proteins. These adducts consist of covalent modifications which occur through the formation of Schiff bases or through Michael addition reactions: (1) Schiff base formation on primary amine (lysine residue) through the reaction between peptides/proteins and 4-HNE, (2) Michael addition of 4-HNE on amino groups (lysine/histidine residues) or thiols (cysteine residue) through the reaction between peptides/proteins and 4-HNE, and (3) Protein-protein cross-linking through the reaction between 4-HNE with histidine and lysine residues from different peptides/proteins.

**Figure 4:**
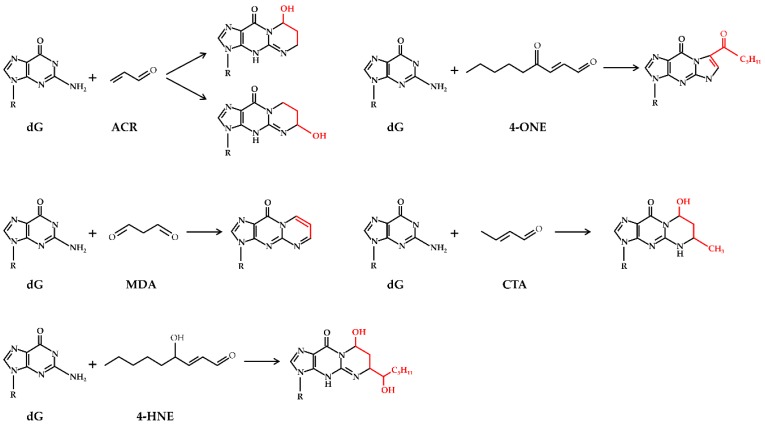
Hypothetical DNA adducts produced by reactive aldehydes.By reacting with DNA, in particular with the deoxyguanosine nucleobases, several reactive aldehydes such as ACR, MDA, 4-HNE, 4-ONE and CTA produce DNA modifications named exocyclic adducts that alter the DNA structure and, if not correctly repaired, may produce carcinogenic effects.

Some of these compounds are known to contribute to the pathogenesis of several diseases, such as atherosclerosis, rheumatoid arthritis, neuropsychiatric disorders, heart disease, cellular reperfusion injury, cancer, and metabolic disorders such as diabetes and hepatic diseases [4,5,7,12,54]. Reactive aldehydes are a group of electrophilic molecules with different features: some of them are very unstable, characterized by a short half-life, while others are long-lived and highly reactive. In the past years, the endogenous formation of reactive aldehydes has drawn great interest. The ability of aldehydes to easily diffuse across biological membranes [55], and to form adducts with macromolecules such as phospholipids, nucleic acids and proteins [7,46,56,57,58], is of particular concern. Adducts consist of covalent modifications, which involve the formation of Schiff bases or Michael addition reactions. To this regard, the reactive aldehydes toxicity against peptides and proteins is due to their ability to alter their structure and/or function through the formation of cross-links between different amino acid chains, thus potentially leading to the production of aberrant protein aggregates (Figure 3) [59]. Concerning the toxicity of reactive aldehydes against DNA, it has been shown that these compounds may react against nucleobases, among which the most affected is guanine, due to its chemical structure prone to oxidative modifications. The most studied DNA modifications caused by reactive aldehydes are the exocyclic adducts (Figure 4) [57,58,60].

4-HNE and 4-ONE are generated from lipid peroxidation of ω-6 PUFAs (e.g., arachidonic acid and linoleic acid) [61]. Among reactive aldehydes, 4-HNE is the most studied, and its toxic effects can be explained by its ability to form protein adducts by reacting with thiols and amino groups of cysteine, histidine, and lysine amino acid residues [62]. For a detailed explanation of the main 4-HNE-modified proteins, see the following publication [63]. 4-ONE is an electrophilic compound that reacts both in vitro and in vivo with nucleobases, in particular with 2′-deoxyadenosine and 2′-deoxycytidine [64,65,66,67].

Unlike 4-HNE and 4-ONE, 4-HHE is generated from ω-3 PUFAs (e.g., docoshexaenoic acid, eicosapentaenoic acid and linolenic acid) and, because of its chemical structure, it is considered a soft electrophil with a lower reactivity compared to 4-HNE [7].

MDA, which is widely used as a marker of lipid peroxidation [68], contains at least two unsaturations [7] and is generally produced by PUFAs. Regarding its toxicity, MDA modifies target proteins through the formation of Schiff base complexes, which occur on the amino groups of lysine, histidine, arginine, glutamine, and asparagine amino acid residues as well as on the *N*-terminal of peptide chains [69]. For a detailed explanation of the main MDA-modified proteins, see the following publication [63]. Moreover, in vitro mutagenicity of MDA has been observed by several authors using the Salmonella tiphimurium assay [70,71,72]. Several studies showed the presence of both MDA and MDA-protein adducts in rheumatoid arthritis patients compared to healthy controls [73,74,75,76]. Moreover, high levels of circulating autoantibodies against MDA-modified epitopes have been detected in serum or plasma of patients affected by rheumatoid arthritis [77,78,79]. CTA or 2-butenal is a carcinogenic aldehyde formed by lipid peroxidation, which is also commonly found in air pollution, in cigarette smoke and in other combustion processes. CTA is able to form adducts with DNA [80,81,82,83] and proteins. In accordance with in vitro mutagenesis assay with Salmonella typhimorium, CTA is a mutagenic compound [84] able to induce hepatocellular carcinoma in rats [85]. About protein modifications, CTA reacts preferentially with lysine and histidine amino acid residues, thus forming β-substituted butanal adducts [86].

ACR or propenal is a metabolite of PUFAs lipid peroxidation, but it is also a ubiquitous environmental pollutant by-product derived by incomplete combustion of organic matter and plastic, cigarette smoke, overheated cooking oils, as well as by anticancer treatment with cyclophosphamide [87,88]. Among reactive aldehydes, ACR is the strongest electrophile, which shows a high reactivity with cysteine, histidine, and lysine amino acid residues. Moreover, ACR forms cyclic adducts with nucleosides in vitro, and is recognized as a potent mutagen [89].

Despite their harmful properties, growing evidence has also demonstrated the hormetic effects of reactive aldehydes [51,63,90,91,92,93]. The term “hormesis” refers to a highly conserved and dose-dependent response of biological systems in which low doses of noxious stimuli activate an adaptive response that increases the functionality and/or resistance of the systems to more severe stress. Conversely, high doses of noxious stimuli cause inhibition or detrimental effects [94]. To this regard, low levels of reactive aldehydes may modulate cell signaling, cellular proliferation and many other processes [7,61,89,95]. A typical example is represented by 4-HNE, which may also act as a signaling molecule by modulating the activity of different stress-related transcription factors, such as nuclear factor-erythroid 2-related factor 2 (Nrf2), activating protein-1, NF-κB, and peroxisome proliferator-activated receptors (PPARs) [96,97,98,99,100]. Moreover, low levels of 4-HNE may stimulate the activity of protein kinase C (PKC), may increase cell proliferation, and the expression of cyclooxygenase type 2 (COX-2) and prostaglandin E2 (PGE2) [51].

## 3. The Endocannabinoid System: Endocannabinoids, Their Lipid Analogues, and the Receptors

Over the last years, the ECS has attracted considerable attention as a signaling system because of its emerging regulatory functions in health and disease.

Several components jointly make up the ECS, and they specifically consist of (1) the ECs, endogenous bioactive lipid mediators generated in the brain and in several peripheral tissues; (2) two membrane G-protein-coupled receptors (GPCRs) referred to as CB1 and CB2, and others, not yet identified, receptors; and (3) several proteins implicated in the biosynthesis, release, transport, and degradation of these lipid mediators [101].

*N*-arachidonoyl-ethanolamine or anandamide (AEA) and 2-arachidonoyl-glycerol (2-AG), both derived from the arachidonic acid, are the best characterized members of the main families of ECs (*N*-acylethanolamines (NAEs) and monoacylglycerols (MAG), respectively) and exert their biological effects by interacting with CB1 and/or CB2 receptors [102]. AEA, an endogenous eicosanoid derivative isolated from pig brain in 1992, was the first EC to be identified [103], and it is well known to modulate several physiological functions being present in the autonomic and in the CNS as well as in the gastrointestinal tract and in the cardiovascular, immune and reproductive systems [104,105].

The second EC ligand to be discovered was 2-AG [106], which has been identified in brain and reproductive tissues in higher concentrations compared to AEA [107,108,109]. Moreover, 2-AG has also been found in the heart, endothelial cells and circulating cells such as macrophages and platelets [104].

Even though AEA and 2-AG interact with both CB1 and CB2 [110], they show different affinity and efficacy. In particular, depending of on the specific tissue, AEA can be either a partial or a full agonist of CB1, whereas it shows a low overall efficacy for CB2, for which it is a relatively weak ligand [111]. On the contrary, 2-AG appears to be a full agonist of both receptors [112] showing higher CB1 and CB2 efficacy than AEA.

Unlike what has been thought for many years, CB1 expression is not restricted to the brain, where it represents the most abundant of all GPCRs [113,114], but it has been also identified, albeit at much lower concentrations, in various peripheral tissues and cell types including adipose tissue, liver, skeletal muscle, kidney, bone, pancreas, myocardium, human coronary artery endothelial and smooth muscle cells and inflammatory cells (macrophages, lymphocytes) [104,115,116].

In the brain, CB1 is widely present in cerebral cortex, hippocampus, caudate-putamen, substantia nigra pars reticulata, globus pallidus, entopeduncular nucleus, and cerebellum [117]. Interestingly, accumulating evidence supports a new mechanism of action of CB1 signalling in the brain, since it has been found in mitochondria, where it probably modulates neuronal energy homeostasis [118]. On the other hand, the CB2, also known as the “immune cannabinoid receptor”, is primarily expressed in immune and hematopoietic cells. However, its presence has also been established at lower, although functionally relevant, levels in the brain, liver, gut, exocrine and endocrine pancreas, reproductive cells, bone, myocardium, human coronary endothelial and smooth muscle cells, and inflammatory cells (e.g., lymphocytes, macrophages, neutrophils) [104,115,119].

CB1 and CB2 are seven-transmembrane-domain proteins both coupled with G_αi/o_ proteins, which inhibit adenylyl cyclase (AC) leading to a reduced protein kinase A (PKA) and PKC activity and to the consequent inhibition of voltage-gated Ca^2+^ channels and activation of inwardly rectifying K^+^ currents [120]. Furthermore, through a common pathway mediated by G_αo_ proteins, CB1 and CB2 are also able to modulate Ras-related protein (Rap) (a member of the Ras small G protein family) and, in particular, it has been postulated that the activation of G_αo_ would release Rap1 guanosine triphosphatase (GTPase) activating protein (Rap1 GAP), which then would be free to inhibit the activity of Rap [121]. Moreover, several observations demonstrated that, depending on the CB1 agonist, this receptor could also interact with G_αs_ proteins [122,123].

On the basis of the cell type, the signaling of CB1 and CB2 may also involve G protein independent mechanisms, leading to the activation of mitogen-activated protein kinases (MAPKs) including p38- and p44/42-MAPKs, c-Jun *N*-terminal kinase (JNK), PKA and PKC, COX-2, and ceramide signaling [124,125,126].

However, beyond binding the CB1 and CB2 there is increasing pharmacological evidence for additional receptor targets for ECs [127], such as the transient receptor potential vanilloid 1 (TRPV1) [127,128,129], the PPARs family [130,131] and the orphan G protein-coupled receptors 119 (GPR119), 55 (GPR55) and 18 (GPR18) [132]. TRPV1 is a member of the vanilloid transient receptor potential cation channel subfamily, abundantly expressed in the cardiovascular system, peripheral nervous system, CNS and in epithelial cells of the bladder and the gastrointestinal tract. It is known to act by activating PKA and the endothelial nitric oxide synthase (eNOS), thus stimulating the production of •NO and the release of calcitonin gene-related peptide and substance P [133,134], which, in turn, lead to the altered ion permeability [135].

The finding that some pharmacological actions of AEA can be mediated by the activation of TRPV1 suggests the capability of this endogenous lipid compound to act as an “endovanilloid” [136,137], although AEA induces typical TRPV1-mediated effects with a lower affinity compared to CB1 [127].

PPARs are a family of transcription factors constituted by three different isoforms (α, β/δ, and γ), widely expressed in tissues with a higher oxidative capacity such as the cardiovascular system and, in particular, cardiomyocytes, endothelial cells, and vascular smooth muscle cells [104], but also in several brain areas and in peripheral tissues such as kidney and liver [138].

After being activated by a ligand, PPARs stimulate gene expression by creating heterodimers with the retinoid X receptor (RXR), thereby binding to specific peroxisome proliferator response elements (PPREs) in the promotor region of target genes [139]. They are involved in different biological processes, such as energy homeostasis, lipid and lipoprotein metabolism, cell proliferation and inflammation, blood pressure control and hypertensive-related complications, such as stroke and renal damage [140,141]. Furthermore, among the different members of the PPARs family, PPAR-α is recently attracting great attention for its anti-oxidative properties [142].

Moreover, AEA has been shown to exert anti-inflammatory and analgesic actions, and to control feeding behavior by activating the isoform α and γ of PPARs receptors [130,143,144]. Unlike AEA, 2-AG has no affinity for TRPV1 and is only able to activate PPARs [144,145].

As above mentioned, additional GPCRs were suggested to participate in non-CB1/CB2-mediated actions of ECs including the GPR18, GPR119 and GPR55 [146]. 

The GPR18, widely expressed in the cardiovascular system, CNS, spleen, and testis, is coupled with G_αi/o_ proteins whose activation results in the AC inhibition and in the modulation of the PI3K/Akt and extracellular signal-related kinases (ERK 1/2) pathways [104]. The G_αs_ coupled-GPR119, primarily expressed in human and rodent pancreas, foetal liver, gastrointestinal tract and in rodent brain, stimulates AC leading to increased intracellular adenosine 3′,5′-cyclic monophosphate (cAMP) levels, thus regulating incretin and insulin hormone secretion [147].

Finally, the GPR55, which is expressed in human brain and liver, but also in rat spleen, vasculature, intestine, foetal tissues, decidua, and placenta, is coupled with G_α12/13_ proteins and increases intracellular Ca^2+^ via the activation of RhoGTPase nucleotide exchange factors (RhoGEFs) [148].

Different from the classical neurotransmitters, the ECs are not stored in intracellular vesicles but are synthesized “on demand” from membrane phospholipid precursors in response to stimuli that trigger an increase in intracellular Ca^2+^ levels [131], and then released from postsynaptic neurons to act on presynaptic CB1/CB2 through a retrograde mechanism [149,150]. However, recent findings suggested that AEA could be stored inside the cell into adiposomes, which are thought to connect plasma membrane to internal organelles along the metabolic route of this EC [151].

Although 2-AG and AEA are both derived from arachidonic acid, they do not share the same anabolic and catabolic enzymes [126]. Depending on the available precursors and the distinct physiological or pathological conditions [131], AEA can be synthesized by multiple routes. The main pathway for AEA biosynthesis consists of the enzymatic cleavage of the precursor *N*-acyl-phosphatidylethanolamine (NAPE), which is mediated by the NAPE-phospholipase D (NAPE-PLD) [152], whereas the biosynthesis of 2-AG begins with the hydrolysis of 2-arachidonoyl-phosphatidylinositol that occurs through the activity of diacylglycerol lipase (DAGL) and phospholipase Cβ [153].

ECs have a short duration of action, being rapidly metabolized by intracellular enzymes such as fatty acid amide hydrolase (FAAH), the main enzyme responsible for AEA degradation [154,155,156], and monoacylglycerol lipase (MAGL), which favors 2-AG catabolism [157].

Additional oxidative enzymes, including COX-2, LOXs and cytochrome P450 may also play a role in the metabolism of both AEA and 2-AG by transforming them in bioactive eicosanoids [158,159], which may activate cannabinoid receptor-independent mechanisms [160].

Beyond the ECs, several other endogenous mediators have attracted considerable attention, despite some of them showed poor affinity for CB1 and CB2 [126]. Among them, palmitoylethanolamide (PEA), stearoylethanolamide (SEA), and oleoylethanolamide (OEA), belonging to the family of NAEs, are the best characterized. However, other lipid analogues have recently been discovered and include *N*-arachidonoyldopamine (NADA), Cis-9,10-octadecanoamide (oleamide or ODA), and *N*-arachidonoylglycine (NAGly) [161], commonly referred to as endovanilloids because of their ability to activate TRPV1. Additionally, 2-arachidonoylglyceryl ether (noladin ether, 2-AGE), O-rachidonoylethanolamine (virodhamine), and arachidonoyl-l-serine (ARA-S) have also been identified [105].

Although still debated, NAEs are generally thought to be cannabinoid-receptor inactive, and they appeared to be responsible for enhancing AEA activity through the so-called ‘‘entourage effect”, which consists in the inhibition of FAAH leading to an increase of AEA tissue levels [162].

PEA and OEA, shorter and fully saturated analogues of AEA, are well-documented high affinity PPAR-α and TRPV1 endogenous ligands and have been shown to exert roles in many physiological and pathological conditions such as satiety, inflammation, pain and memory consolidation [163,164,165,166,167,168]. Furthermore, due to their high expression in the CNS, growing evidence established their protective effects in neurodegenerative and neuropsychiatric disorders [169,170,171,172]. Moreover, PEA is also an endogenous agonist of GPR55, while OEA can bind GPR119.

As already mentioned, NADA belongs to the endovanilloid class of ECs and is an endogenous ligand of CB1, TRPV1 and PPAR-γ [105]. Since this compound is widely distributed in the brain, particularly in the striatum, hippocampus, cerebellum, and dorsal root ganglia, it has been shown to exert a role in neuronal pain and inflammation [105]. Interestingly, NADA also showed antioxidative and anti-inflammatory effects on glial cells [105].

2-AGE is an endogenous analogue of 2-AG, able to bind to CB1, PPAR-α and very weakly to CB2 [143,173]. Moreover, thanks to its chemical structure, 2-AGE is more stable compared to AEA and 2-AG, which are rapidly hydrolysed in vivo [102].

Virodhamine is the ester of arachidonic acid and ethanolamine and is more expressed in the periphery compared to the brain, where it is rapidly converted to AEA, due to its chemical instability. Virodhamine has been shown to act as a full agonist of CB2 and a partial agonist of CB1, whereas at higher concentrations it can be also a CB1 antagonist [174]. Furthermore, it appeared to activate also PPAR-α [143] and GPR55 [175].

NAGly is an efficacious ligand of the orphan GPR18, with no CB1, CB2, or TRPV1 activity, and shows analgesic, anti-inflammatory, and vasorelaxant properties [176].

AraS is another ECs-like compound structurally similar to AEA, which was demonstrated to produce endothelium-dependent arterial vasodilatation and to activate p44/42 MAPKs in cultured endothelial cells, effects also observed after ECs treatment [105]. To date, AraS has been shown to be a low efficacy agonist to GPR18 without binding CB1/CB2 or additional ECs receptors [105].

Lastly, ODA is a full agonist of cannabinoid receptors with selectivity for the CB1, whose activation is the primary responsible for ODA effects [105].

As suggested by the wide range and distribution of the cannabinoid receptors and by the several compounds that take part in the ECS, the latter is now considered as a complex signaling system that may play a key role in physiological and pathological conditions. Thus, targeting these intricate pathways can represent a challenge in finding a therapeutic benefit for cannabinoid-based drugs in various disorders.

## 4. Modulation of Oxidative Stress and Lipid Peroxidation through Cannabinoid Receptors by Endocannabinoids and Their Lipid Analogues

It is well documented that there is an important cross-talk between the ECS and various redox-dependent processes. Indeed, the ECS has been reported as a novel therapeutic target against free radical-induced lipid peroxidation. In fact, it has been shown that ECS is implicated in the development of a growing number of diseases linked with redox homeostasis deregulation, including those associated with metabolic disorders, such as type 2 diabetes and obesity, cardiovascular diseases, as well as various neuropsychiatric and neurodegenerative disorders, ischemia/reperfusion (I/R) injury, and renal diseases [2,4,5,54,177].

In the past decade, various and complex pathways have been studied to clarify the role of ECs in the modulation of redox imbalance, whose knowledge is the specific aim of this review.

There is accumulating evidence that shows the ability of ECs to alter the expression and/or the activity of enzymes implicated in the generation of these reactive small molecules (such as NOX2 and NOX4), and to modulate the production of cellular ROS/RNS by controlling mitochondrial-derived ROS/RNS generation [177].

Alternatively, ECs and their lipid analogues may modulate oxidative stress and lipid peroxidation either by conveying beneficial free radical scavenging effects or through targeting CB1 and CB2 [8,9,10]. Furthermore, CB1 and CB2 are differentially involved in oxidative stress modulation. In fact, several studies highlight that the activation of CB1 results in a redox imbalance enhancement, whereas CB2 stimulation is responsible for lowering ROS/RNS formation [9]. The beneficial or detrimental effects of ECs may be cell- and injury-type-specific and may depend on the stage of the disease progression as well [8].

This aspect was further investigated by Han and colleagues, who demonstrated a different role of CB1 and CB2 in regulating macrophage activity, and, in particular, the former appeared to be directly involved in the induction of intracellular ROS/RNS formation with consequent pro-inflammatory macrophage response, while the latter, after being activated by AEA, was able to negatively regulate CB1-stimulated ROS/RNS generation, through a pathway involving the small G protein, Rap1 [9]. The authors further showed that blocking CB1 while selectively activating CB2 might suppress pro-inflammatory responses of macrophages.

These data are consistent with other studies using cisplatin-induced renal dysfunction [178,179,180,181], in which it was observed that blocking the CB1 [179], or activating the CB2 [180,181], led to the attenuation of the cisplatin-induced increase of renal 4-HNE and ROS/RNS-generating enzymes (NOX2 and NOX4) expression, thus protecting against tubular damage.

**Figure 5:**
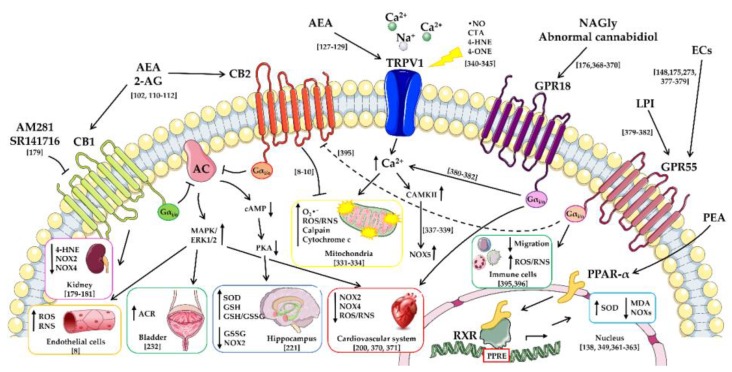
Role of endocannabinoids (ECs) and their lipid analogues in modulating reactive oxygen and nitrogen species (ROS/RNS) and reactive aldehydes formation. AM281: 1-(2,4-dichlorophenyl)-5-(4-iodophenyl)-4-methyl-N-4-morpholinyl-1H-pyrazole-3-carboxamide; SR141716: rimonabant; CB: cannabinoid receptors; AEA: anandamide; 2-AG: 2-arachidonoyl-glycerol; TRPV: transient receptor potential vanilloid; CTA: crotonaldehyde; NAGly: *N*-arachidonoylglycine; GPR18: G protein-coupled receptor 18; GPR55: G protein-coupled receptor 55; LPI: l-α-lysophosphatidylinositol; ECs: endocannabinoids; PEA: palmitoylethanolamide; PPARs: peroxisome proliferator-activated receptors; SOD: Cu^2+^/Zn^2+^-superoxide dismutase; MDA: malondialdehyde; PPRE: peroxisome proliferator response element; RXR: retinoid X receptor; NOX: NADPH oxidase enzyme; GSH: glutathione; GSSG: oxidized glutathione; ACR: acrolein; MAPK/ERK1/2: mitogen-activated protein kinases/extracellular signal-regulated kinases; PKA: protein kinase A; cAMP: adenosine 3′,5′-cyclic monophosphate; CAMKII: Ca^2+^/calmodulin-dependent protein kinase; AC: adenylyl cyclase.

Other examples of the opposite effects of CB1 and CB2 come from studies conducted in animal models of obesity and type 1 and 2 diabetes mellitus, where an increase of oxidative stress is observed [182,183,184]. In fact, in these models, increased levels of ECs in various renal cells contribute to the development of oxidative stress, as a result of renal CB1 activation, whereas inhibition of CB1 or activation of CB2 are able to ameliorate such effects (Figure 5) [185].

Overall, the over activation of the ECS that occurs in many type of tissue injury may induce oxidative stress, inflammatory cell infiltration, and the consequent cell death through CB1 activation [8,179], while it may also serve as an endogenous compensatory mechanism to limit early inflammatory response and interrelated oxidative stress-cell death through the activation of CB2 [186].

Interestingly, a cross-talk between redox homeostasis and ECS is particularly involved in the regulation of the cardiovascular system and metabolic tissues (i.e., liver, skeletal muscle and adipose tissue) [187,188], where CB1 and CB2 are widely distributed. Furthermore, previous studies have suggested increased ECs levels in many cardiovascular disorders, such as cardiomyopathies, atherosclerosis, and hypertension [189].

It is well known that cardiovascular diseases are associated with oxidative stress, which leads to the accumulation of lipid peroxidation-derived reactive aldehydes and may consequently cause an increase in the formation of ROS/RNS and/or a decrease in the antioxidant defense [2].

In this regard, it has been demonstrated that, after being activated by AEA, CB1 expressed in endothelial cells [190] and in cardiomyocytes in a murine model of doxorubicin-induced cardiomyopathy [8], induce the activation of the p38-JNK-MAPK pathway and increase the generation of ROS/RNS. These effects lead to cell death and resulted to be partially attenuated by the pharmacological inhibition of CB1 [9].

In contrast to CB1, the activation of CB2 appeared to exert cardioprotective effects by reducing O_2_•^−^ production and decreasing endothelial cell activation. These findings are in agreement with recent studies showing that CB2 activation, by ECs and their analogue lipid mediators, protects against oxidative stress-induced tissue damage in experimental models of I/R injury [191,192,193,194,195], cardiovascular inflammation, and/or atherosclerosis [191,196,197].

Among the cardiovascular diseases, atherosclerosis is due to altered homeostatic redox processes with progressive ROS/RNS over production, which leads to the generation and deposition of toxic oxidized low-density lipoproteins (oxLDL) in the vessel wall. It has been clearly demonstrated that OxLDL promote the activation of NOXs and the synthesis of O_2_•^−^ by a cluster of differentiation 36 (CD36) scavenger receptor-mediated method, effects that can be counteracted by several compensatory mechanisms including the involvement of the ECS [198].

Support for this comes from the observation that increased production of O_2_•^−^ and enhanced NOXs activation in atherosclerosis correlated with increased rates of 2-AG biosynthesis in the vessel wall, which may be a compensatory response to oxidative stress via CB2 signaling [199].

In agreement with these results, it has been observed that the genetic disruption of CB2 in Apolipoprotein E-deficient mice (ApoE^−/−^), a murine model of atherosclerosis, is the cause of boosted O_2_•^−^ generation, whereas its stimulation reduced vascular O_2_•^−^ release, resulting in the suppression of ROS/RNS generation and a subsequent reduction in the size of atherosclerotic lesions (Figure 5) [200].

Further evidence of the protective effects of ECs in atherosclerosis comes from the demonstration that CB1 inhibition in ApoE^−/−^ mice is able to promote the down-regulation of vascular angiotensin II type 1 receptor (AT1), which is responsible for NOXs activation when stimulated by angiotensin II [201]. Consequently, the decreased expression of AT1, mediated by CB1 inhibition, leads to the reduction of NOXs activity and oxidative stress, thereby improving endothelial function and exerting beneficial direct vascular effects [201].

Since the discovery that the levels of NAEs are higher in several pathological conditions linked with redox homeostasis impairment, these compounds are attracting great attention as a survival response toward oxidative damage [202].

Indeed, it has been clearly shown that NAEs, particularly 16:0 and 18:0, exert protective effects in many diseases by the inhibition of free radical-induced lipid peroxidation [203], which is considered one of the main causes of cell damage and death [204].

In particular, previous findings discovered an involvement of two long-chain NAEs, PEA and SEA, in the inhibition of lipid peroxidation in liver mitochondria membranes of acute hypoxic hypoxia animal model [203], a pathological condition associated with an increase in partially reduced oxygen products, which represent the main cause of lipid oxidation-induced formation of reactive aldehydes [205]. The authors suggested that the inhibitory effect of NAEs on lipid peroxidation depends on the length of acyl chain and is related to their ability to protect membranes [206].

These results are in good agreement with other data showing that OEA treatment of rat heart mitochondria is able to reduce the production of MDA, which is one of the end products of lipid peroxidation in cell membrane [203].

Among NAEs, OEA, PEA, and AEA appeared to inhibit Cu^2+^-induced in vitro lipid peroxidation in plasma lipoproteins [202] and cardiac mitochondria [207], consequently showing antioxidant properties in the pathogenesis of atherosclerosis. Moreover, Zolese and collaborators demonstrated that, depending on its concentration of incubation, PEA exerts both anti-oxidative and pro-oxidative effects on radical-induced oxidation of plasma LDL [208]. The authors showed that higher PEA concentrations could be responsible for its pro-oxidant effect, whereas PEA at lower levels is able to suppress reactive aldehydes, generated by lipid peroxidation, and to decrease the consumption rate of LDL endogenous anti-oxidants, thereby showing anti-oxidant properties [208].

In the context of cardiovascular diseases is also interesting to mention hypertension, which is characterized by (1) deregulation of ECS with increased activity of FAAH and MAGL, (2) increased levels of AEA, 2-AG, and NADA, and (3) increased expression of CB1 [209], effects that are accompanied by an imbalance of redox homeostasis (decreased activities of glutathione peroxidase (GPx), glutathione reductase (GR) and the antioxidant enzymes Cu^2+^/Zn^2+^-superoxide dismutase (SOD) and catalase (CAT)).

It has been demonstrated that increased levels of AEA, following chronic administration of the FAAH inhibitor URB597 in a rat model of hypertension [210], significantly enhanced the expression of the CB1, thus preventing the hypertension-induced decrease of SOD, glutathione (GSH) and glutathione transferase (GT) activities and consequently lowering ROS generation and inducing hypotension. However, it has been postulated that the enhanced AEA levels are responsible for the perturbation of membrane phospholipid metabolism resulting in PUFAs chain cyclization or fragmentation. This causes an increase in the formation of α,β-unsaturated reactive aldehydes such as 4-HNE, MDA, and 4-ONE in the liver of hypertensive rats [209].

It is well documented that ECS and oxidative stress may also play a role in the pathophysiology of liver diseases [188,211]. For instance, DeLeve and collaborators [212] reported that CB1 activation is responsible for liver inflammation and, therefore, induces non-alcoholic liver disease, whereas the CB2 stimulation appeared to have protective effects in liver damage through reducing liver oxidative stress [213].

Accumulating evidence supports the involvement of ECS as a therapeutic potential in many neurodegenerative pathologies such as Alzheimer’s and Parkinson’s diseases, in which oxidative stress has been recognized as one of the hallmarks of the pathology [4,171,172,214,215,216,217].

Indeed, the brain is a tissue with a high oxygen consumption whose cell membranes are particularly rich in PUFA side-chains and, therefore, highly sensitive to lipid peroxidation and oxidative damage [54,183,218].

NOXs enzymes have been shown to be significant sources of ROS/RNS during tissue injury and, in particular, it has been observed that the activation of NOX2 contributes to oxidative imbalance–induced CNS damage [219], while its inhibition is able to ameliorate cerebral oxidative stress injury [220].

A recent study conducted by Jia and collaborators defined AEA as a promising candidate for the treatment of oxidative stress–related neurological disorders [221]. In particular, AEA has been found to protect a mouse hippocampal neuron cell line from H_2_O_2_-induced redox imbalance by increasing SOD and GSH intracellular levels, reducing oxidized glutathione (GSSG), increasing the GSH/GSSG ratio, and lowering NOX2 expression. All of these effects were completely abolished by both CB1 antagonist administration and CB1-siRNA, suggesting that the ability of AEA to ameliorate oxidative stress in hippocampal neurons may be mediated by CB1 activation (Figure 5) [221].

Similarly, it has been also reported that the stimulation of CB1 is able to reduce intracellular ROS/RNS generation and NOX2 expression thus enhancing nigrostriatal dopaminergic neurons survival in a mouse model of Parkinson’s disease [222].

These findings supporting the beneficial effects of CB1 activation against ROS/RNS formation in the brain seem to be controversial in comparison to what above mentioned for the cardiovascular and renal tissues. An explanation for this argument comes from growing evidence suggesting that the pathways underlying the interplay between cannabinoid receptors and oxidative stress modulation may be cell type–specific [177].

Notably, as well as responses mediated by CB1, further data showed that the modulation of CB2 signaling, either by using specific CB2 agonists [223,224,225] or by inhibiting 2-AG degrading enzyme MAGL [226], can ameliorate the morphological changes induced by oxidative stress and attenuate cerebral β-amyloid plaque accumulation in a mouse model of Alzheimer’s disease carrying mutated human APPswe and PS1dE9 genes [227,228].

Interestingly, in vitro studies revealed that a selective CB1 agonist, arachidonyl-2-chloroethylamide, decreased the Fe^2+^-induced lipid peroxidation in the brain, through a metal-chelating mechanism, as well as the •OH radicals generated by the Fenton system [229].

Moreover, the activation of the recently discovered mitochondrial CB1 by arachidonyl-2-chloroethylamide has been demonstrated to reduce oxidative stress, thereby exerting neuroprotective effects in I/R injury [227]. To this regard, CB2 activation also appeared to have a role in attenuating I/R damage through lowering ROS/RNS production and lipid peroxidation [227].

The involvement of CB2 in I/R injury has also been investigated in a context of propofol cardioprotection in an in vivo model of myocardial I/R injury, in which it has been observed that CB2 inactivation reverses propofol cardioprotective and anti-oxidative effects [230]. These findings imply that the enhancement of ECs release and the subsequent activation of CB2 signaling are responsible for the reduced oxidative stress mediated by propofol cardioprotection in myocardial I/R injury [230].

Furthermore, CB2 are expressed in the bladder [231] and are involved in the treatment of hemorrhagic cystitis, a common side effect of Cyclophosphamide, an antineoplastic alkylating agent usually metabolized by the liver to ACR, which is accumulated in urine and therefore is considered to be the main responsible for Cyclophosphamide-induced cystitis [232]. The findings of this study revealed that, following stimulation, CB2 attenuated ACR-induced cystitis through modulating ERK1/2 MAPK pathways (Figure 5) [232].

AEA and 2-AG are also involved in the progression of cancer, where they were shown to exert protective effects against increased ROS/RNS production–induced tumor [233], leading to apoptosis in normal and cancer cells by modulating ERK and ROS/RNS pathways [234].

## 5. Modulation of Oxidative Stress and Lipid Peroxidation through the Transient Receptor Potential Vanilloid Channels by Endocannabinoids and Their Lipid Analogues

The transient receptor potential (TRP) channels superfamily is a wide group of tetrameric channels formed by six transmembrane domains and a cation-selective pore. On the basis of its amino acid sequence homology, TRP superfamily, in mammals, is organized into six subfamilies, which include TRP canonical, TRP melastatin, TRP ankyrin, TRP mucolipin, TRP vanilloid, and TRP polycistin channels. TRP channels are ubiquitously expressed in most mammalian cells [235,236] and they depolarize cells by altering membrane potential or intracellular Ca^2+^ concentration. With the exception of some TRP channels, most of them are non-selective and weakly voltage-sensitive [237]. TRP channels are fundamental players of sensory physiology as they respond to environmental stimuli such as taste, light, sound, smell, touch, temperature, and osmolarity [238]. Today, only a few endogenous ligands are known to activate TRP channels, and it is not yet clear how they are activated in vivo [237]. However, several experiments performed on knockout mice are revealing the complexity and the different functions of TRP channels [238,239,240].

In this review, we will focus mainly on the vanilloid TRP (TRPV) channels subfamily and how they respond to oxidative stress and lipid peroxidation-induced cell damage. Currently, six TRPV channels (TRPV1-6) have been identified and divided into two subgroups: TRPV1-4 and TRPV5-6, based on their amino acid sequence, functions, and cation selectivity. A detailed review on TRPV channels pharmacology has been provided by Vriens and colleagues [241]. Briefly, TRPV1 is expressed in primary sensory neurons, in few brain regions (hypothalamus, intrafascicular, supramammillary and rostral raphe nuclei, entorhinal cortex, hippocampus, and periaqueductal gray), as well as in smooth muscle cells of several thermoregulatory tissues (skin, dura, tongue, trachea, cremaster muscle, and ear) [242]. TRPV1 seem to be activated by heat above 43 °C, by low pH [243,244,245], by vanilloid compounds (e.g., capsaicin and capsinate) [243,246], by ethanol [247,248], as well as by several endogenous compounds such as AEA [127], OEA [249], NADA [250], *N*-oleoyldopamine (OLDA) [251], and arachidonic acid-derived metabolites released by LOXs [252]. Moreover, TRPV1 activity is modulated by various intracellular molecules and signals including calmodulin [253,254], ATP [255], phosphatidylinositol 4,5-bisphosphate (PIP2) and phosphatidylinositol 3,4,5-trisphosphate (PIP3) [256], PKC [257], PKA [258], as well as protein phosphatase calcineurin [259].

Among the main functions, in addition to acting as a thermoreceptor, TRPV1 regulates the normal functioning of urinary bladder [260], controls the gut afferent sensitivity to distension and acids [261] and it also allows the taste perception of sodium chloride [262]. From a physiopathological point of view, TRPV1 has a direct role in the behavioral response to ethanol [247,248,263], as well as in inflammatory airway diseases [264]. Moreover, TRPV1 is also involved in vascular dementia as well as in Huntington’s disease, where its activation promotes neuroprotection, increase learning and memory, and reduce oxidative stress [265,266,267].

Differently, TRPV2 is a weakly Ca^2+^-selective channel, which seems to be activated by thermal stimuli above 53 °C but not by low pH or vanilloid compounds [268]. TRPV2 is expressed in different tissues including brain, spinal cord, spleen, and intestine, as well as in vas deferens, bladder, heart, kidney [269], and immune cells such as monocytes and dendritic cells [270]. It is noteworthy that TRPV2 signaling plays an important role in the endosomal pathway, where TRPV2 modulates the fusion between endosomal membranes by releasing Ca^2+^ from early endosomes [271,272] as well as in phagocytosis [273,274].

TRPV3 is a non-selective cation channel activated by temperatures of 33–39 °C, which showed a marked sensitization following repeated heat stimuli [275,276]. Moreover, TRPV3 could be activated by several vegetable-derived molecules, such as eugenol, thymol, camphor and carvacrol [277,278]. Furthermore, other agents such as PIP2/PIP3, calmodulin, ATP, and inflammatory mediators like histamine, bradykinin, and PGE2 are able to sensitize TRPV3 function [278,279,280,281]. Moreover, it was hypothesized that, in rodent skin cells, heat-induced TRPV3 signaling could mediate an autonomous response to heat stimulation, thus acting as thermoreceptors in keratinocytes [275,282]. In support of this evidence, TRPV3 knock-out mice showed strong deficits in response to heat stimulation [277]. Likewise, TRPV4 is also activated by heat, in particular by temperatures of 27–34 °C, as well as by osmotic and mechanical stimuli [283,284]. Among putative endogenous ligands, it was observed that AEA, 2-AG, and arachidonic acid indirectly activate TRPV4 by epoxyeicosatrienoic acids released from cytochrome P450 epoxygenases [285,286]. As for TRPV1 and TRPV3, TRPV4 activity is modulated by PIP2/PIP3, calmodulin and ATP [287,288,289] and by several protein kinases, such as PKA, PKC, Src family kinases (SFKs), and serum glucocorticoid-induced protein kinase-1 (SGK1) [290,291,292,293]. TRPV4 channels are widely expressed in epithelial cells of the renal convoluted tubule, trachea, submucosal glands, as well as in neutrophils, in autonomic nerve fibers, in peripheral sensory ganglia, in hair cells of the inner ear, and brain structures such as vascular organ of the lamina terminalis and the hypothalamic median preoptic region [283,294,295]. Due to its widespread expression, TRPV4 is involved in several physiological functions. In particular, it mediates temperature sensation in skin keratinocytes, anterior hypothalamus, and sensory ganglia [275,283,284]. TRPV4 is also involved in mechanosensation [296] and contribute to the normal functioning of the urinary bladder [297,298] and pulmonary alveoli [299,300] and to the development of mechanical hyperalgesia in inflammatory states [301].

TRPV5 and TRPV6 share a high sequence homology (74% of identity) and form highly Ca^2+^-selective channels, which are not activated by heat [302,303,304]. As for the other TRPV family members, the activity of TRPV5 and TRPV6 is modulated by a variety of second messengers, including Ca^2+^, Mg^2+^, ATP, PIP2, calmodulin, and PKC [302,303,305,306,307,308,309,310,311,312,313]. TRPV5 is expressed in several tissues but is mostly abundant in renal tubules, where it regulates transcellular transport and reabsorption of Ca^2+^ [314]. Furthermore, TRPV5 is also involved in bone remodeling [315,316]. TRPV6 is widely expressed [305,317,318] but is mostly distributed in the intestine, kidney, and placenta, where it respectively modulates the Ca^2+^ transcellular entry, reabsorption, and transfer to fetus [319,320,321,322].

Among endogenous ligands of TRPV, or endovanilloids, there are leukotriene B4 and 12-hydroperoxyeicosatetraenoic acid that belong to the eicosanoid family, produced by lipoxygenase-mediated oxidation of PUFAs (especially arachidonic acid), which are potent activators of TRPV1 [252,323]. Other lipid-derived mediators of TRPV are epoxyeicosatrienoic acids, such as 5′,6′-epoxyeicosatrienoic acid, which are synthesized from arachidonic acid by cytochrome P450 epoxygenases and may activate TRPV1 and TRPV4 [286,324].

As AEA is structurally similar to arachidonic acid as well as to PUFAs, it can be metabolized by COX-2 and LOXs. In particular, COX-2 converts AEA into prostaglandin-ethanolamides, which are endoperoxide molecules also known as prostamides [325,326]. On the other hand, LOXs convert AEA into hydroperoxy fatty acids, such as 12- and 15-hydroperoxyeicosatetraenoylethanolamide, which are, respectively, synthesized by 12-LOX and 15-LOX [327,328]. In guinea-pig bronchi, these oxidized lipid mediators seem to act as TRPV1 agonists and are also responsible, at least partially, for the contractile action of AEA [329].

Growing evidence supports a key role for TRPV, especially TRPV1, in the modulation of oxidative stress and lipid peroxidation mediated by endocannabinoids, their lipid analogues, and other lipid-related mediators. As known, AEA is considered an endovanilloid because of its ability to activate TRPV1 [127,136,330]: several in vitro analyses performed on human and rat cell lines have shown that AEA induces apoptotic effects via a TRPV1-mediated mechanism, which induces and increase in intracellular Ca^2+^ levels, mitochondrial uncoupling, oxidative stress due to increased O_2_•^−^ formation, cytochrome c release as well as calpain and caspase-3 activation [331,332,333]. Similarly, another in vitro study performed on human bladder cancer T24 cells showed that TRPV1 activation by capsaicin was correlated in a dose-dependent manner with an increase of cytosolic Ca^2+^ levels, with mithocondrial membrane depolarization and a marked ROS/RNS generation, which reduced T24 cells viability (Figure 5) [334].

Other studies showed that AEA was able to increase ROS/RNS production by targeting TRPV1, [335,336], which lead to the activation of the Ca^2+^/calmodulin-dependent protein kinase II (CAMKII), and to the upregulation of NOX5 [337,338,339].

Moreover, it was observed, in the human esophageal epithelial cell line Het1A, that acid- or capsaicine-induced activation of TRPV1 leads to an increased production of intracellular ROS/RNS levels as well as to increased ROS/RNS- or HNE-modified proteins. In the same study, immunoprecipitation analyses of 4-HNE-stimulated Het1A cells revealed, also, that TRPV1 was modified by 4-HNE [340]. In addition to 4-HNE, TRPV1 is directly activated by •NO, oxidants and other chemical agents through the modification of cysteine free sulfhydryl groups [341]. Moreover, functional assays with mutated TRPV showed that cysteine residues 553 and 558, between the fifth and sixth transmembrane domains, are essential for •NO-induced activation of TRPV1, TRPV3, and TRPV4 and thus are potential targets of nitrosylation [342]. In addition, TRPV1 nitrosylation by •NO increased the intracellular Ca^2+^ levels and thus enhanced the channel sensitivity to H^+^ and heat. These sensitizing effects induced by nitrosylation of cysteine residues were further supported by the use of oxidizing agents such as diamide and chloramine-T [343]. Furthermore, several studies reported that TRPV1 is also responsive to other electrophilic compounds generated during oxidative stress. To this regard, in TRPV1 channel-expressing human embryonic kidney (HEK) cells, a modest TRPV1 activation was observed following 4-ONE treatment (100 µM) [344]. Another TRPV1 activator is CTA. In particular, an in vitro study performed on murine cardiomyocytes incubated with CTA showed an increase in TRPV1 and NOXs levels, in ROS/RNS formation, in apoptotic events, and a decrease in the activity of mithocondrial proteins such as aconytase, uncoupling protein 2, and peroxisome proliferator-activated receptor-gamma coactivator-1alpha [345].

## 6. Modulation of Oxidative Stress and Lipid Peroxidation through the Peroxisome Proliferator-Activated Receptors-Alpha by Endocannabinoids and Their Lipid Analogues

Because of the high expression of PPAR-α in kidney, liver, heart, and brain, it is well documented that the activation of these transcription factors exerts protective roles in cardiovascular as well as renal, hepatic, and neurodegenerative diseases [138,346,347,348,349].

There is rising acknowledgment that the beneficial effects of PPAR-α stimulation could be explained by its ability to dampen oxidative stress in several pathological conditions linked to the redox impairment. A number of reports point to the involvement of various mechanisms through which PPAR-α agonists can modulate antioxidants.

In particular, the identification of PPREs elements in promoter regions of CAT and SOD genes in rat [347] additionally supported the involvement of these nuclear receptors in lowering ROS/RNS formation and lipid peroxidation products.

Nevertheless, PPAR-α is not only involved in suppressing ROS/RNS generation, but it can also play a role in modulating enzymes involved in ROS/RNS synthesis and/or scavenging. Consistently, the decrease in striatal SOD expression, which resulted in the 6-hydroxydopamine (6-OHDA)-induced Parkinson disease mouse model, was completely counteracted by PPAR-α agonists confirming the ability of this nuclear receptor to regulate the transcription of antioxidant enzymes (Figure 5) [138,346,350].

For instance, Diep and colleagues reported that the PPAR-α -induced suppression of oxidative stress in cardiovascular diseases is mediated by the ability of PPAR-α activators to inhibit angiotensin II-induced activation of NOXs in the vascular wall [348] and to increase scavenging enzymes as well.

Among the PPAR-α ligands, ECs and their lipids analogues have been shown to play a prominent role in affecting redox homeostasis in several oxidative stress-related pathologies, through a PPAR-α dependent mechanism. Consistently, it has been shown that PPAR-α stimulation by PEA lowers blood pressure and prevents hypertension-induced renal damage in hypertensive rats by inhibiting the subunit p47phox of NOXs (a key regulatory subunit essential for NOXs functioning) [349], and by significantly reducing the hypertension-induced increased levels of MDA in urine and renal tissues (Figure 5) [348].

Moreover, through PPAR-α activation, PEA appeared to simultaneously enhance the antioxidant defense by increasing SOD expression in the kidney [348], thus protecting from renal damage. In agreement with these results, other studies further support the potential beneficial effects of PEA activated-PPAR-α on kidney diseases [351]. For instance, it has been demonstrated that PEA, by targeting PPAR-α, is able to prevent kidney damage induced by I/R injury through dampening the lipid peroxidation products in the kidney, thereby leading to a reduction of neutrophil recruitment [352].

Moreover, because of the high expression of PPAR-α and its endogenous lipid agonists in the CNS, it has been demonstrated that PPAR-α activation can exert neuroprotective properties in several neuropathological conditions, especially in neurodegenerative disorders [169], by modulating the redox balance that resulted altered in these situations.

Further support for this comes from the observation that the brain areas that display the highest PPAR-α expression exhibit an overlapping expression pattern with key enzymes involved in ROS/RNS synthesis and/or scavenging including CAT, SOD1 and acyl-CoA oxidase 1 (ACOX1) [353,354,355], whose genes are known to be under the control of PPAR-α [356,357].

Thanks to its anti-oxidative properties, PPAR-α protects against normal brain aging and regulates the onset and progression of neurodegenerative disorders [358,359]. Interestingly, evidence suggests that in conditions of neurodegeneration, oxidative stress itself is responsible for the induction of PPAR-α expression. As a matter of fact, in hippocampal CA1 pyramidal cells of a transgenic mouse model of Alzheimer’s disease, an increase in the levels of PPAR-α simultaneously with the production of ACR and 8-hydroxy(de)oxyguanosine, which represent markers of oxidative imbalance, was observed [360]. Such increase in hippocampal PPAR-α expression could trigger the induction of its target genes encoding for peroxisomal membrane protein-70 (PMP70) and ACOX1, which are involved in fatty acyl-CoA transport across peroxisomal membranes and peroxisomal β-oxidation respectively, by evoking a compensatory response to Aβ-mediated mitochondrial insult that occurs in early stage of Alzheimer’s disease [4,5,6,360].

In this context, PEA was demonstrated to protect neurons and glia from oxidative stress by reducing MDA formation, thereby restoring a proper cellular redox state, and this effect appeared to be PPAR-α-dependent [171,172,361,362]. It has also been established that PEA neuroprotective effects are mediated, at least in part, through the de novo synthesis of neurosteroids (particularly allopregnanolone), which is triggered by PPAR-α activation [362].

The abovementioned findings, coupled with a recent report demonstrating that PEA treatment (through binding PPAR-α) is able to induce SOD and dampen ROS/RNS-induced oxidative damage in 6-OHDA-induced mouse model of Parkinson disease, additionally suggest the neuroprotective scavenging effects of this lipid compound (Figure 5) [363]. Beyond the ECs, several other synthetic ligands of PPAR-α have been shown to exert antioxidative properties. For instance, Wy14643 through binding PPAR-α is able to protect rabbit hearts from I/R injury by increasing the expression of the oxidative stress-inducible isoform of heme oxygenase and to preserve hippocampal neurons from H_2_O_2_ challenge by modulating mitochondrial fusion and fission events [360].

Moreover, it should be noted that the production of PPAR-α endogenous ligands, PEA and OEA as the mostly characterized, could be differently affected by physiological and pathological oxidative stress-related conditions. For instance, the ROS/RNS metabolism imbalance, which is responsible for oxidative stress-induced brain aging and neurodegeneration, can quantitatively and qualitatively modify the production of PPAR-α agonists and thus differently modulate PPAR-α-mediated pathways in neuronal and astroglial cells [169].

Additionally, the interplay between PPAR-α and oxidative-stress-induced lipid peroxidation comes also from the observation that NOXs activated-4-HNE is able to act as an endogenous PPAR-α activator leading to the discovery of the so called “lipid peroxidation products–PPARs–NOXs axis” [364]. The regulation of this axis, which represents an alternative pathway mediating ROS/RNS production, could ensure additional strategies to counteract oxidative-stress-related disorders.

## 7. Modulation of Oxidative Stress and Lipid Peroxidation through Other Receptors by Endocannabinoids and Their Lipid Analogues

Recently, in addition to PPAR-α and TRPV1, the orphan receptors GPR18, GPR55 and GPR119 were assessed as novel cannabinoid-related receptors [365]. Structurally, GPRs are GPCRs and, among them, GPR18, GPR55 and GPR119 share a limited primary sequence homology with CB1 and CB2.

GPR18 was discovered for the first time in 1997 by Gantz and colleagues [366]. GPR18 is widely expressed in testis and spleen, and in lesser extent in several other tissues such as thymus, lymph nodes, peripheral blood leukocytes, small intestine, and appendix, thus suggesting a regulatory role for GPR18 in the immune system [366]. Moreover, GPR18 was also found in several brain regions such as hypothalamus, brainstem, cerebellum, and striatum as well as in lung, thyroid and ovary [367]. Several studies reported that NAGly is the endogenous ligand of GPR18 that induces an elevation of intracellular Ca^2+^ levels [176]. The same authors demonstrated also that GPR18 activation was pertussis toxin-sensitive, suggesting the involvement of a G_αi/o_ protein in this response [176]. Despite these first evidence, several authors reported variable responses of GPR18 following the administration of NAGly [368,369].

For the first time Penumarti and colleagues demonstrated that GPR18 is expressed in the rostral ventrolateral medulla of rats and exerts tonic restraining influence on blood pressure [370]. In particular, authors observed that the systemic administration of abnormal cannabidiol, a synthetic agonist of GPR18, induced a dose-dependent reduction of blood pressure and increased heart rate. In addition, GPR18 activation increased neuronal adiponectin and •NO, and finally reduced neuronal ROS/RNS levels. These findings suggested for the first time a sympathoinhibitory role of GPR18 (Figure 5) [370].

More recently, another study confirmed that chronic GPR18 activation with its agonist abnormal cannabidiol produced hypotension, suppressed the cardiac sympathetic dominance, and improved left ventricular function in conscious rats [371]. In the same study, ex vivo analysis of plasma, heart, and vascular tissues of treated rats revealed an increase in cardiac and plasmatic adiponectin levels, an increase in aortic eNOS expression, augmented levels of vascular and serum •NO, high levels of myocardial and plasmatic guanosine 3′,5′-cyclic monophosphate (cGMP), an increase of myocardial Akt and ERK1/2 phosphorylation, and, more importantly, reduced myocardial ROS/RNS formation [371]. These results suggest a protective role of GPR18 in cardiovascular diseases, in particular highlights the possibility to consider GPR18 as a viable molecular target for developing new antihypertensive drugs which are able to improve also the cardiac function.

Human GPR55 receptor was identified for the first time in 1999, through in silico studies, and was subsequently cloned [372]. GPR55 receptor is widely expressed, and therefore its activity was correlated with multiple physiological processes. In particular, GPR55 is expressed in the frontal cortex, striatum, hippocampus, hypothalamus, cerebellum, and brainstem [372,373]. Moreover, GPR55 was also found in peripheral organs and cells such as dorsal root ganglion [148], spleen, adrenal glands, jejunum, ileum [373], pancreas [374], bones [375] and microglia [376]. The GPR55 pharmacology and its downstream signaling are not yet certain. Nevertheless, some authors reported that ECs such as AEA, 2-AG, and virodhamine can activate both etherologous and native GPR55-expressing cells [148,273,377], while other groups reported that ECs are weak ligands [378,379], may act as partial agonists [175], or are not able to activate GPR55 receptors [380,381]. Another open debate regards the ability of PEA to activate [373] or not the GPR55 receptors [148,382]. Despite the controversial results about the ability of ECs to activate GPR55, it is well accepted that the endogenous lipid l-α-lysophosphatidylinositol (LPI) and its analogue 2-arachidonoyl-sn-glycero-3-phosphoinositol are endogenous ligand of GPR55 [379,380,381,382]. However, it is necessary to specify that LPI is not selective only for GPR55 [383]. Moreover, GPR55 may also heterodimerize with other receptors, such as CB2 [384], thus further confounding the results obtained so far.

About the mechanisms of downstream signaling, GPR55 activation was associated with an increase of intracellular Ca^2+^ levels, with the activation of RhoA and ERK1/2 pathway, and with the activation of several transcription factors, such as the nuclear factor of activated T-cells and the cAMP response element binding protein (CREB) [380,382].

The human orphan receptor GPR119 was identified for the first time in 2003 by sequence alignment tools analysis [385]. GPR119 is expressed mainly in pancreas and gut, in particular in β-cells and pancreatic polypeptide-producing PP cells, where its activity modulates the glucose-dependent insulin secretion [386,387], as well as in enteroendocrine L-cells, where it regulates the secretion of glucagon-like peptide 1 [388,389]. GPR119 is also expressed in liver [390] and skeletal muscle [Cornall et al., 2013]. In normal-weight and healthy patients it was observed that gut GPR119 expression rapidly increased following acute fat exposure [391], thus suggesting a potential involvement of GPR119 in type 2 dyabetes, metabolic disorder, and obesity.

The main endogenous ligands of GPR119 are, in order of potency, OLDA, OEA, PEA, and AEA [392,393]. Other endogenous GPR119 agonists are 2-oleoylglycerol [394] and oleoyl-lysophosphatidylcholine [386]. Clearly, also in this case, further studies are required to better characterize the pharmacological profile of GPR119.

Increasing evidence suggests that ECs may regulate ROS/RNS levels and thus reactive aldehydes formation by targeting GPR55. In this regard, Balenga and colleagues showed that GPR55 activity modulates RhoA-dependent neutrophil migration, and it may prevent oxidative damage [395]. In particular, this study, performed on neutrophils, demonstrated that 2-AG-induced ROS/RNS production, which was mediated by a CB2-dependent mechanism, appeared to be significantly decreased following the co-treatment with the GPR55 agonist LPI [395]. This negative interaction between GPR55 and CB2 was observed during neutrophil respiratory burst. Therefore, after an initial synergism in inducing chemotaxis, GPR55 and CB2 disengaged and, by a functional repression, GPR55 decreased CB2-induced oxidative damage by blocking CB2 downstream signaling [395]. Conversely, a recent study performed on human natural killer cells and monocytes unveiled a proinflammatory role of GPR55 activation (Figure 5) [396], which could be potentially correlated with an increase of ROS/RNS production and thus with oxidative stress.

## 8. The Role of Antioxidant System as Scavenger of ROS/RNS and Reactive Aldehydes

The “endogenous antioxidant system” relies on several enzymes, peptides, cofactors, and other molecules that are essential for the maintenance of a physiological redox homeostasis. Overall, endogenous antioxidants may be divided into two main groups, formed by enzymatic and non-enzymatic antioxidants [6,397]. The enzymatic group include CAT [398], SOD [399,400], GPx, GR, GT [401], thioredoxin (Trx) and thioredoxin reductase (TrxR) [402] while the non-enzymatic group include several antioxidant molecules such as GSH, GSSG, [403], vitamin A (retinol) [404], vitamin C (l-ascorbic acid) [405], vitamin E (tocopherols) [406], coenzyme Q10 (CoQ10) [407], carotenoids [408], flavonoids, polyphenols [409,410,411], minerals such as Se^2+^ [412], Cu^2+^, and Zn^2+^ [413], as well as metabolites such as uric acid, bilirubin [414] and melatonin [415], which also possess antioxidant properties.

Briefly, CATs are Cu^2+^/Zn^2+^-dependent enzymes present in peroxisomes that catalyze the conversion of H_2_O_2_ in water and oxygen [398]. Among SOD enzymes, cytolosic SOD are Cu^2+^/Zn^2+^-dependent enzymes, while mitochondrial SODs are Mn^2+^-dependent enzymes that metabolize O_2_•^−^ into H_2_O_2_ and oxygen. Therefore, SOD represents the first line of defense against reactive aldehydes formation [400]. GPx, GR and GT are Se^2+^-dependent enzymes that, together with GSH and GSSG, constitute the glutathione system, which contributes to eliminate H_2_O_2_ and other reactive molecules [403]. Similarly, Trx, TrxR, and NADPH constitute the thioredoxin system, which is critical for redox regulation of protein function and signaling via thiol redox control [402].

Vitamin A is produced in the liver, derives from β-carotene and acts as a lipid peroxidation blocker by preventing the chaining process in the propagation phase [404]. Similarly, also vitamin E acts as a lipid peroxidation blocker by donating a hydrogen atom to peroxyl radicals, thus forming tocopheroxyl radicals which are unable to continue the propagation phase of lipid peroxidation [416]. Vitamin C is effective in scavenging several ROS/RNS as well as in the detoxification of peroxyl and hydroxyl radicals [405]. CoQ10 is involved in the neutralization of the damages induced by peroxyl radicals and also in the regeneration of vitamin E [407]. Uric acid is known to prevent protein nitrosylation, as well as lipid and protein peroxidation, and therefore it is considered as a protectant agent of the CNS [417]. Melatonin is a natural scavenger derived from tryptophan, which is involved in the neutralization of several ROS/RNS and thus reduces the generation of reactive aldehydes [415]. Finally, flavonoids and polyphenols are ubiquitous plant-derived molecules, which act as chelators and scavengers of ROS/RNS as well as of hydroxyl and peroxyl radicals [418,419].

## 9. Conclusions

Oxidative stress represents an underlying disturbance that is involved in many pathophysiological conditions. Increasing evidence suggests that tissues with a high oxygen consumption, such as brain and heart among others, are particularly sensitive to lipid peroxidation products and free radical accumulation, which are responsible for oxidative stress–induced damages with consequent cell death [2,4,5,6,218].

Thus, acting on the cellular processes that suppress the generation of these reactive small molecules or altering the expression and/or activity of enzymes involved in their formation may be crucial for the treatment of a growing number of diseases linked with redox homeostasis deregulation.

In this scenario, there is rising acknowledgment about a cross-talk between the ECS and various redox-dependent processes. Indeed, it has been observed that the redox impairment induces the enhancement of AEA and 2-AG levels, as a consequence of phospholipid hydrolysis [420,421], and the upregulation of CB1 and CB2 expression [422,423], as well as the downregulation of FAAH [422].

A large number of reports point to the involvement of ECs and their lipid analogues in regulating ROS/RNS and reactive aldehydes generation through targeting CB1 and CB2 [8,9,10] and thereby exerting protective effects in cardiovascular as well as renal, hepatic, neuropsychiatric, and neurodegenerative diseases.

Moreover, it has been observed that, depending on the type of cell and/or injury, cannabinoid receptors show opposite effects in oxidative stress modulation, since CB1 activation results in a redox imbalance enhancement, while CB2 stimulation is responsible for lowering oxidative stress [9,223] and may convey beneficial free radical scavenging effects.

Overall, the mechanisms by which CB2 receptors, following ECs-mediated activation, are involved in the reduction of oxidative injury seem to be primarily mediated by the reduction of NOX2 and NOX4, and the simultaneous induction of the antioxidant defense through the increase of the SOD scavenging enzymes [180,181].

Emerging evidence indicates that the neuroprotective, cardioprotective and renoprotective effects of ECs and NAEs are additionally mediated by CB1/CB2-independent mechanisms and involve the contribution of alternative intracellular targets such as PPAR-α, TRPV1, GPR55, and GPR18 [169,348,349,370,371,395].

In particular, an interplay between PPAR-α and oxidative stress has been suggested from the observation that an imbalance in the redox state may modulate several signaling pathways, including PPAR-α signaling, via transcriptional regulation and post-translational modification.

Among the PPAR-α ligands, PEA appeared to exert beneficial effects by simultaneously enhancing the antioxidant defense through the increase of SOD expression and inhibiting NOXs activity with a consequent reduction of the lipid peroxidation products such as MDA [138].

Although the huge amount of knowledge has been gained about the effects of the ECs on oxidative stress and lipid peroxidation in several pathological conditions, many ECS compounds fail during clinical trials due to inefficacy or unforeseeable safety concerns. For the treatment of the cardiovascular diseases, for instance, no cannabinoid-based drugs have been approved so far, except for those acting as PPARs agonists [348]. Among the limitations that play a role in restricting the translation of ECs studies into clinical trials, the different animal paradigms as well as the route of administration used (central vs peripheral) and the differences between species seem to be primarily involved.

Moreover, most of the studies have focused on the role of CB1, CB2, TRPV1, PPARs and less is known about other candidates such as GPR18, GPR55 and GPR119.

Despite promising goals have been achieved over the last years on ECS research, there is an urgent necessity to expand the knowledge on the ECs complex signalling in order to better identify an explanation of the serious side-effects observed in clinical studies. Lessons from clinical experience should encourage the scientific community to better clarify how to modulate the ECS thus leading to major breakthroughs in the treatment of many diseases.

Overall, the findings discussed in this review may further elucidate the complex interaction existing between ECS, oxidative stress, and lipid peroxidation, resulting in a better understanding of the multiple beneficial effects of this signaling system in several pathological conditions related to a redox status impairment.

## Abbreviations

•NO = nitric oxide; •OH = hydroxyl radical; ^1^O_2_ = oxygen singlet; 2-AG = 2-arachidonoyl-glycerol; 2-AGE = 2-arachidonoylglyceryl ether or noladin ether; 4-HHE = 4-hydroxy-hexanal; 4-HNE = 4-hydroxy-2-nonenal; 4-ONE = 4-oxo-nonenal; 6-OHDA = 6-hydroxydopamine; AC = adenylyl cyclase; ACOX1 = acyl-CoA oxidase 1; ACR = acrolein; AEA = *N*-arachidonoyl-ethanolamine or anandamide; ApoE^−/−^ = Apolipoprotein E-deficient mice; ARA-S = arachidonoyl-l-serine; AT1 = angiotensin II type 1 receptor; CAMKII = Ca^2+^/calmodulin-dependent protein kinase II; cAMP = adenosine 3′,5′-cyclic monophosphate; CAT = catalase; CB1 = cannabinoid receptor type 1; CB2 = cannabinoid receptor type 2; CD36 = cluster of differentiation 36; CGD = chronic granulomatous disorder; cGMP = guanosine 3′,5′-cyclic monophosphate; CNS = central nervous system; CoQ10 = coenzyme Q10; COX-2 = cyclooxygenase type 2; COXs = cyclooxygenases; CREB = cAMP response element binding protein; CTA = crotonaldehyde; DAGL = diacylglycerol lipase; dG = deoxyguanosine; ECS = endocannabinoid system; ECs = endocannabinoids; eNOS = endothelial nitric oxide synthase; ERK1/2 = extracellular signal-related kinases; FAAH = fatty acid amide hydrolase; GPCRs = G-protein-coupled receptors; GPR18 = G protein-coupled receptor 18; GPR55 = G protein-coupled receptor 55; GPR119 = G protein-coupled receptor 119; GPx = glutathione peroxidase; GR = glutathione reductase; GSH = glutathione; GSSG = oxidized glutathione; GT = glutathione transferase; GTPase = guanosine triphosphatase; H_2_O_2_ = hydrogen peroxide; HClO = hypochlorous acid; HEK = human embryonic kidney; I/R = ischemia/reperfusion; JNK = c-Jun *N*-terminal kinase; LOXs = lipooxygenases; LPI = l-α-lysophosphatidylinositol; MAG = monoacylglycerols; MAGL = monoacylglycerol lipase; MAPKs = mitogen-activated protein kinases; MAPK/ERK1/2 = mitogen-activated protein kinases/extracellular signal-regulated kinases; MDA = malondialdehyde; NADA = *N*-arachidonoyldopamine; NAEs = *N*-acylethanolamines; NAGLy = *N*-arachidonoylglycine; NAPE = *N*-acyl-phosphatidylethanolamine; NAPE-PLD = NAPE-phospholipase D; NF-κB = nuclear factor-κB; NO_2_ = nitric dioxide; NO_2_^−^ = nitrite; NO_3_^−^ = nitrate; NOS = nitric oxide synthase; NOS1 = NOS type 1; NOX2 = NOX type 2; NOX3 = NOX type 3; NOXs = NADPH oxidase enzymes; Nrf2 = nuclear factor-erythroid 2-related factor 2; O_2_•^−^ = superoxide anion; O_3_ = ozone; ODA = Cis-9,10-octadecanoamide or oleamide; OEA = oleoylethanolamide; OLDA = *N*-oleoyldopamine; ONOO^−^ = peroxynitrite; oxLDL = oxidized low-density lipoproteins; PEA = palmitoylethanolamide; PGE2 = prostaglandin E2; PIP2 = phosphatidylinositol 4,5-bisphosphate; PIP3 = phosphatidylinositol 3,4,5-trisphosphate; PKA = protein kinase A; PKC = protein kinase C; PMP70 = peroxisomal membrane protein-70; PPARs = peroxisome proliferator-activated receptors; PPRES = peroxisome proliferator response elements; PUFAs = polyunsatured fatty acids; Rap = Ras-related protein; Rap1GAP = Rap1 GTPase activating protein; RhoGEFs = RhoGTPase nucleotide exchange factors; ROS/RNS = reactive oxygen and nitrogen species; RXR = retinoid X receptor; SEA = stearoylethanolamide; SFKs = Src family kinases; SGK1 = serum glucocorticoid-induced protein kinase-1; SOD = superoxide dismutase; TRP = transient receptor potential; TRPV = transient receptor potential vanilloid; TRPV1 = transient receptor potential vanilloid 1; Trx = thioredoxin; TrxR = thioredoxin reductase.
